# Cerebello-brainstem dominant form of X-linked adrenoleukodystrophy with intrafamilial phenotypic variability

**DOI:** 10.3389/fneur.2022.999419

**Published:** 2022-11-09

**Authors:** Jae-Hwan Choi, Hyun Sung Kim, Eun Hye Oh, Jae Hyeok Lee, Chong Kun Cheon

**Affiliations:** ^1^Department of Neurology, Pusan National University School of Medicine, Pusan National University Yangsan Hospital, Yangsan, South Korea; ^2^Research Institute for Convergence of Biomedical Science and Technology, Pusan National University Yangsan Hospital, Yangsan, South Korea; ^3^Division of Medical Genetics and Metabolism, Department of Pediatrics, Pusan National University School of Medicine, Pusan National University Children's Hospital, Yangsan, South Korea

**Keywords:** adrenoleukodystrophy, *ABCD1*, cerebellar ataxia, very long-chain fatty acids, phenotype

## Abstract

**Objectives:**

This study aimed to describe the clinical and radiological characteristics of a cerebello-brainstem dominant form of X-linked adrenoleukodystrophy (X-ALD).

**Methods:**

Three affected members from a family with cerebellar ataxia received full neurological, laboratory and radiological examinations. Genetic diagnoses were confirmed using whole-exome sequencing and protein structural modeling.

**Results:**

All affected members presented with slurred speech, ataxia, and spasticity, but showed obvious differences in phenotypic severity and radiological findings. The levels of very long-chain fatty acids (VLCFA) were elevated in each member, while only one had adrenal dysfunction. Genetic analysis identified a hemizygous missense mutation (c.887A>G, p.Tyr296Cys) of the ATP-binding cassette subfamily D member 1 gene (*ABCD1*) in all affected members, which is likely to destabilize the overall structure of the ABCD1 protein.

**Conclusions:**

We report a cerebello-dominant form of X-ALD caused by a missense variant in *ABCD1*. This report highlights intrafamilial phenotypic variability in X-ALD.

## Introduction

X-linked adrenoleukodystrophy (X-ALD) is the most common peroxisomal disorder caused by mutations in the ATP-binding cassette subfamily D member 1 gene (*ABCD1*) of Xq28 (1). *ABCD1* encodes a peroxisomal membrane ABC transporter that is responsible for delivering very long-chain fatty acids (VLCFAs) into the peroxisomes for degradation (1, 2). Thus, defects in *ABCD1* lead to impaired peroxisomal β-oxidation and a subsequent accumulation of saturated VLCFAs in the blood and various tissues, including the adrenal cortex and nervous system.

Patients with ALD present various phenotypes depending on the tissues affected (1). Adrenomyeloneuropathy (AMN) and childhood cerebral ALD (CCALD) are the most common phenotypes. AMN is characterized by adult-onset slow progressive spastic paraplegia due to the involvement of the spinal cord and peripheral nerves, while the onset of CCALD occurs during childhood, with rapid neurological deterioration and progressive cerebral demyelination, which lead to a vegetative state within a few years. There are several additional ALD phenotypes, including adult cerebral ALD (ACALD), cerebello-brainstem dominant ALD, and isolated adrenocortical insufficiency (Addison-only). The cerebello-brainstem dominant form, in which the infratentorial structures are mainly involved, has been estimated to account for 1–2% of ALD, but may be underdiagnosed because patients present with progressive cerebellar dysfunction that mimics multiple system atrophy (MSA) or idiopathic late-onset cerebellar ataxia (ILOCA) (3–12). In addition, various phenotypes can be observed even within the same family, making it difficult to diagnose ALD (13–15).

Here, we present a cerebello-brainstem dominant form of X-ALD with diverse clinical and radiological manifestations in a Korean family.

## Materials and methods

### Subjects and clinical evaluation

**Figure 1:**
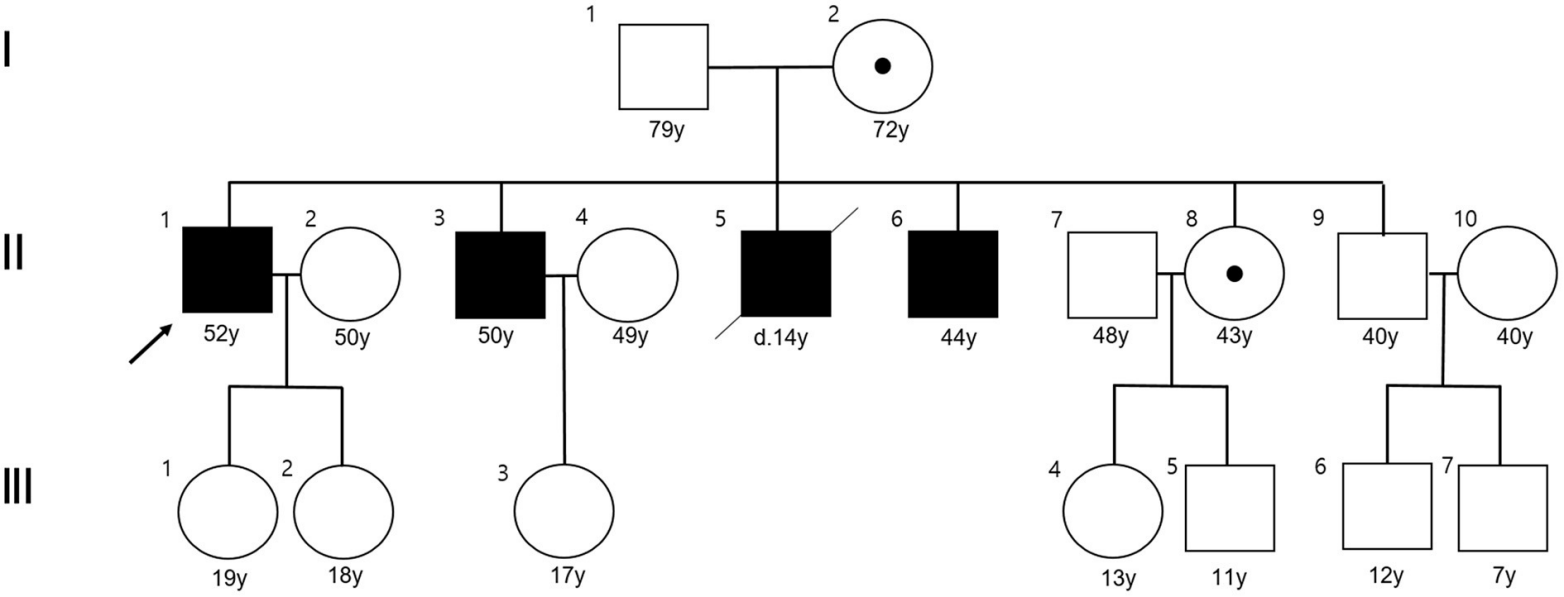
Pedigree of the present family. The proband is indicated by an arrow. Affected males are designated by filled symbols and the carrier female by circle with dot (squares for males and circles for females).

Three members from two consecutive generations of a Korean family with ataxia were enrolled at Pusan National University Yangsan Hospital (Figure 1). Three affected members (proband, II-3, and II-6) received full neurological and neuro-otological evaluations by the authors (H.S.K and J.H.L). Eye movements including nystagmus, saccades, smooth pursuit, and vestibulo-ocular reflex (VOR) were recorded using three-dimensional video-oculography (SLMED, Seoul, Korea). Laboratory evaluations including thyroid and adrenal function, vitamin B12, and paraneoplastic antibody tests, a VLCFA assay, and radiological studies such as brain and spine MRI were performed to determine the etiology of cerebellar ataxia. Adrenal function was evaluated by measuring plasma adrenocorticotropic hormone (ACTH) and cortisol concentrations, and performing a rapid ACTH stimulation test. Nerve conduction studies (NCSs), pure tone audiogram (PTA), and fundus examinations were also conducted.

### Molecular analysis

#### Whole-exome sequencing

Since gene tests for spinocerebellar ataxia types 1–3, 6, 7, and 17, dentatorubropallidoluysian atrophy, fragile X syndrome, and Friedreich's ataxia were all negative, we performed whole-exome sequencing as the next step. Pure genomic DNA was isolated from the peripheral blood leukocytes of the affected members using the QIAamp DNA Blood Midi Kit (Qiagen, Hilden, Germany) according to the manufacturer's protocols. Most of the exonic regions of~22,000 human genes were captured using one of the following three kits, depending on when the patient was enrolled: Agilent Sure Select kit (version C2, December 2018), Twist capture kit (Twist Bioscience, San Francisco, CA, USA), or xGen Exome Research Panel v2 (Integrated DNA Technologies, Coralville, IA, USA). Sequencing was performed using a NovaSeq6000 device (Illumina, San Diego, CA, USA) with 150 bp paired-end reads. The binary base call sequence files generated by the NovaSeq6000 device were converted and demultiplexed to FASTQ files, which were aligned to the human reference genome (GRCh37/19 from NCBI, February 2009) to generate BAM files using BWAMEM (version 0.7.17). Aligned BAM files were sorted and extracted using the samtools stats software (version 1.9). Variant calling files were generated following the GATK Best Practices (GATK version 3.8). The mean coverage depth was 125X (> 20 X = 97%). Variant classification and interpretation were largely based on guidelines recommended by the American College of Medical Genetics and Genomics (ACMG). Mutation nomenclature was based on the cDNA reference sequences for *ABCD1* (NM_000033.4).

#### Validation by Sanger sequencing

Sanger sequencing was used to confirm the causative variants. All causative variants were bi-directionally sequenced using the PRISM BigDye Terminator Kit (version 3.1, Applied Biosystems, Foster City, CA, USA). The sequencing products were resolved on a PRISM 3130XL sequencer (Applied Biosystems) and the chromatograms were analyzed using Sequencher software (version 4.9, Gene Codes, Ann Arbor, MI, USA).

#### Protein structural modeling

Protein structural modeling was performed for *ABCD1* variants. The crystal structures of the wild-type *ABCD1* domains were generated using SWISS-MODEL (https://swissmodel.expasy.org/). Structural images were generated using PyMOL (version 29, https://pymol.org/2/).

## Results

### Clinical analysis

The clinical characteristics of the three affected members are listed in Table 1. Another member (II-5) had progressive gait disturbance and limb weakness from the age of 10 years, and died at 14 years.

**Table 1:** Clinical characteristics of patients with X-linked adrenoleukodystrophy.

	**Proband (II-1)**	**II-3**	**II-6**
Sex/age (years)	Male/52	Male/50	Male/44
Age at onset (years)	50	34	43
Symptoms and signs			
MMSE	25/30	20/30	29–30
Dysarthria	(+)	(+)	(+)
Ocular motor dysfunction	Spontaneous right-beating nystagmus Hypermetric saccades Bilateral saccadic pursuit	(-)	Spontaneous right-beating nystagmus Hypermetric saccades Bilateral saccadic pursuit
Ataxia	(+)	(+)	(+)
SARA score	13.5	16	24
Motor weakness	(-)	(+), left lower limb	(+), both lower limbs
Spasticity	(+)	(+)	(+)
Extrapyramidal symptom	(-)	(+), left hand dystonia	(-)
Sensory disturbance	(-)	(-)	(-)
Urinary dysfunction	(-)	(-)	(+)
Hyperreflexia	(+)	(+)	(+)
VLCFA ratio			
C22:0 (μmol/L, normal range ≤ 96.3)	30.64	40.87	35.65
C24:0 (μmol/L, normal range ≤ 91.4)	64.24	76.93	61.34
C26:0 (μmol/L, normal range ≤ 1.3)	3.96	3.71	3.51
C24:0/C22:0 (normal range ≤ 1.39)	2.10	1.88	1.72
C26:0/C22:0 (normal range ≤ 0.023)	0.129	0.091	0.098
Adrenal impairment	Abnormal result for rapid ACTH test	(-)	(-)
Pure tone audiometry	Right SNHL (mild)	Both SNHL (high frequency)	Right SNHL (mild)
Nerve conduction study	No peripheral neuropathy	No peripheral neuropathy	Distal sensory neuropathy in upper limbs
Brain MRI	T2-weighted hyperintensities around the dentate nuclei (at the age of 52 years)	T2-weighted hyperintensities in the corticospinal tract and middle cerebellar peduncle (at the age of 37 years)	Diffuse T2-weighted hyperintensities in the midbrain, pons, middle cerebellar peduncle and cerebellum (at the age of 44 years)

ACTH, adrenocorticotropic hormone; MMSE, mini-mental state examination; SARA, scale for assessment and rating of ataxia; SNHL, sensorineural hearing loss.

**Figure 2:**
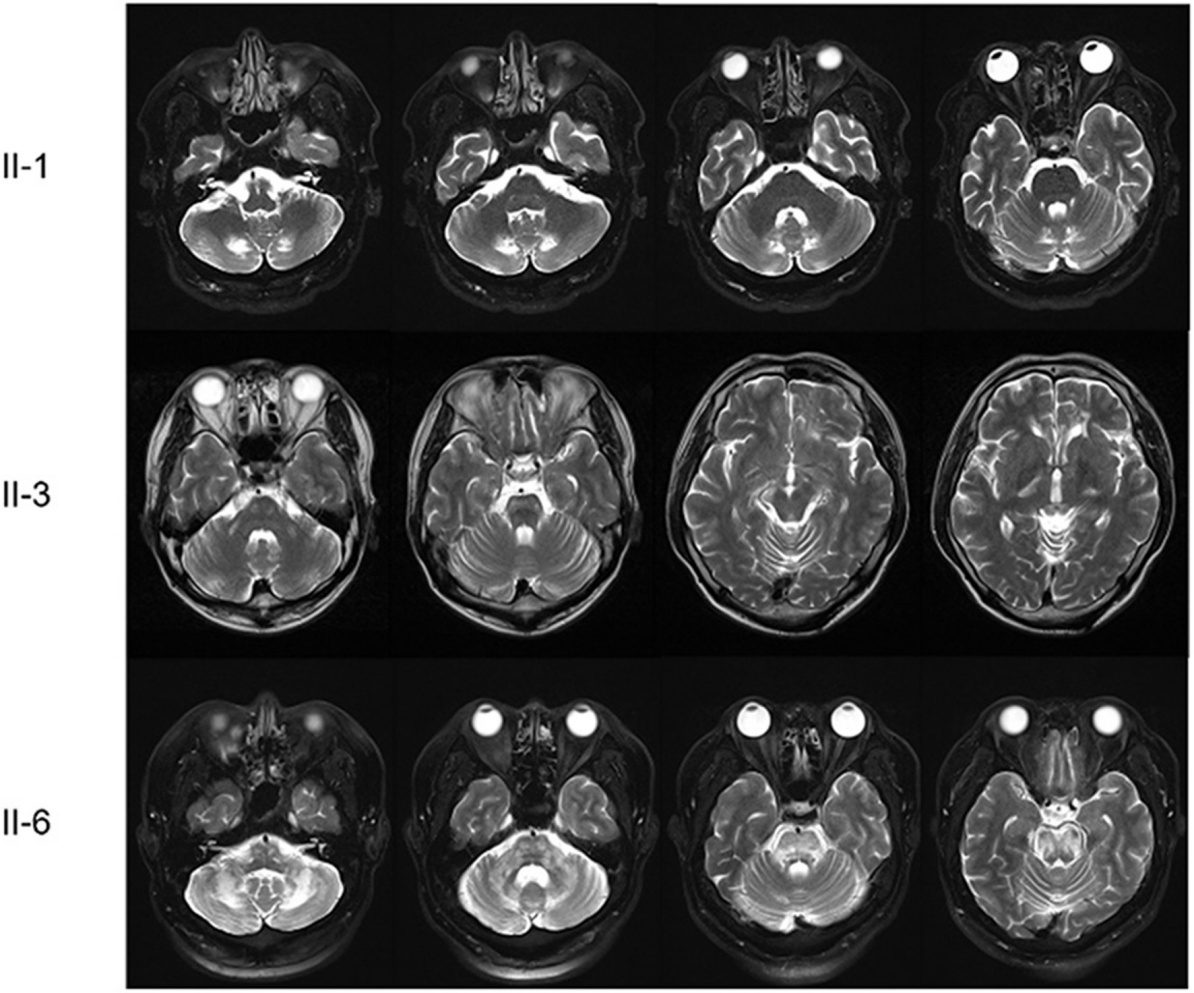
Brain MRI scans of the patients. The proband (II-1) had symmetric T2-weighted hyperintensities in the dentate nuclei and the surrounding cerebellar white matter. The first younger brother (II-3) presented brainstem and cerebellar atrophy with asymmetric T2-weighted hyperintensities in the middle cerebellar peduncles, cerebral peduncles, posterior limbs of the internal capsules, and periventricular white matter of the frontal lobe. The third younger brother (II-6) had diffuse T2-weighted hyperintensities in the midbrain, pons, middle cerebellar peduncle and cerebellum.

The proband was a 52-year-old male who presented with dizziness, unsteadiness and dysarthria for 2 years, with gradual onset and progression. Neurological examinations indicated mild dysarthria and bilateral dysmetria in the upper and lower limbs. He was able to stand and walk without support, but had difficulties in tandem walking. Ocular motor tests revealed spontaneous right-beating horizontal nystagmus, hypermetric saccades, and abnormal smooth pursuit in bilateral horizontal directions, but normal VOR functions. He had bilateral spasticity and hyperreflexia in the lower limbs, but not cognitive impairment, motor weakness, sensory disturbance, or urinary dysfunction. Laboratory evaluations revealed a defective rise of the cortisol level in the ACTH stimulation test, and elevated levels of VLCFAs such as C26:0, and in the C24:0/C22:0 and C26:0/C22.0 ratios. There was a mild degree of right sensorineural hearing loss (SNHL) in PTA. NCS results were normal. Brain MRI showed symmetric T2-weighted hyperintensities in the dentate nuclei and the surrounding cerebellar white matter, but spine MRI results were unremarkable (Figure 2).

The younger brother of the proband (II-3) was a 50-year-old male with a 16-year history of gait disturbance and cognitive impairment. He developed unsteadiness, dysarthria, and left lower limb weakness at the age of 34 years, which gradually progressed over time, and became wheelchair-bound at 37 years. Brain MRI performed at 37 years old revealed brainstem and cerebellar atrophy with asymmetric T2-weighted hyperintensities in the middle cerebellar peduncles, cerebral peduncles, posterior limbs of the internal capsules, and periventricular white matter of the frontal lobe (Figure 2). An initial diagnosis of multiple sclerosis (MS) was made based on the symptoms and MRI findings. He visited our hospital at the age of 50 years, and the Mini-Mental State Examination applied at admission revealed difficulties in maintaining attention, performing calculations, and recalling registered three words. Neurological examinations showed bilateral dysmetria and pyramidal signs including spasticity and hyperreflexia that were severe in the left side. He had also dystonia in the left hand and weakness in the left lower limb. Laboratory evaluations indicated elevated VLCFA levels but normal adrenal function. PTA showed bilateral SNHL, especially at high frequencies. NCS results were normal. At the follow-up MRI performed when he was 49 years old, new white-matter hyperintensities were observed in the bilateral parieto-occipital lobes, but spine MRI results were unremarkable.

Another younger brother (II-6) was a 44-year-old male who presented with a 1-year history of progressive dizziness, unsteadiness, and dysarthria. Neurological examinations showed severe dysarthria and dysmetria in all limbs, and difficulties in standing and walking without support. Similar to the proband, ocular motor tests indicated spontaneous right-beating horizontal nystagmus, hypermetric saccades, and abnormal smooth pursuit. He had bilateral lower limb weakness (MRC grade 4) with spasticity and hyperreflexia, and urinary dysfunction, but not cognitive impairment or sensory disturbance. Laboratory evaluations revealed elevated VLCFA levels but normal adrenal function. PTA indicated a mild right SNHL, and the NCS revealed distal sensory neuropathy in the upper limbs. Brain MRI showed diffuse T2-weighted hyperintensities in the midbrain, pons, middle cerebellar peduncle and cerebellum, while spine MRI results were unremarkable (Figure 2).

With patients only taking Lorenzo's oil for several months, it is still difficult to find improvements in neurological symptoms.

### Molecular and structural impact of the variant proteins related to X-ALD

**Figure 3:**
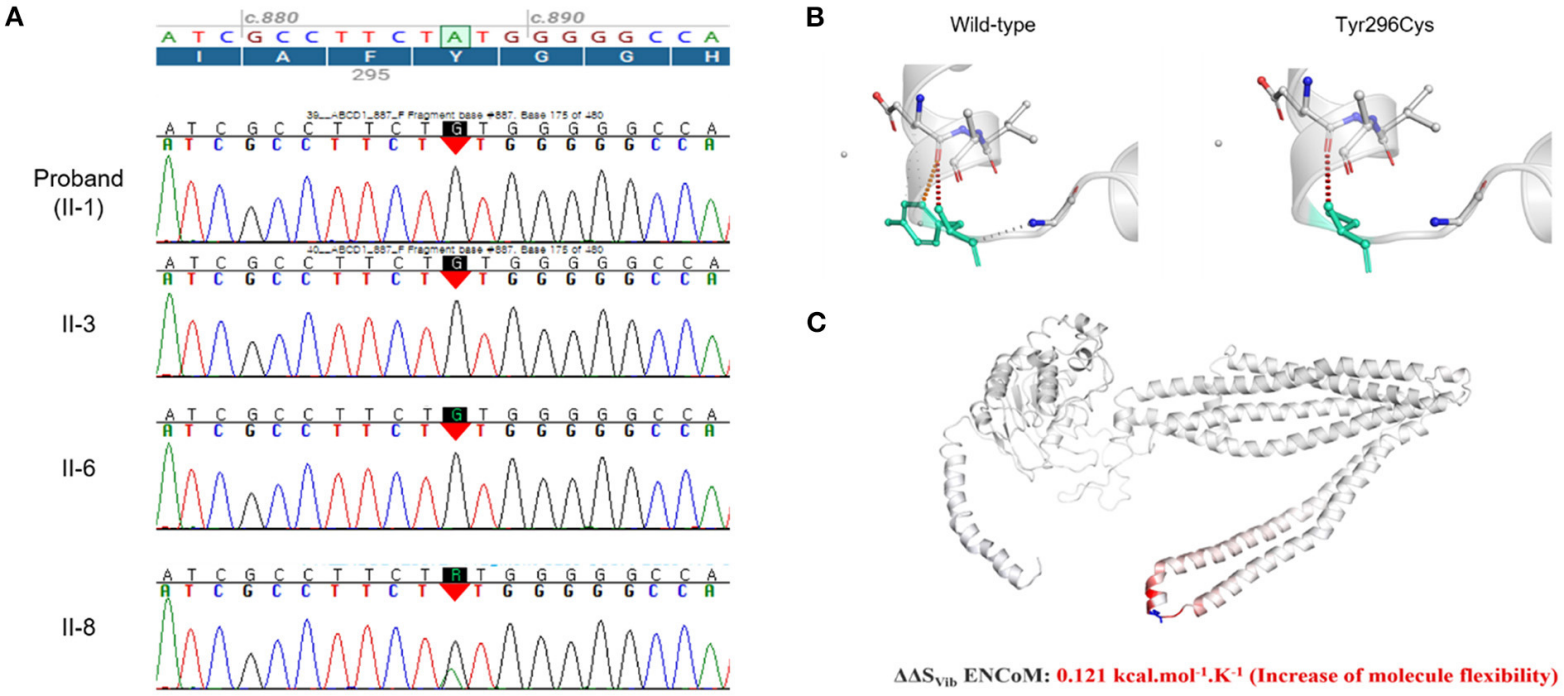
**(A)** Sanger sequencing confirmed a hemizygous missense variant (c.887A>G, p.Tyr296Cys) of the ATP-binding cassette subfamily D member 1 gene (*ABCD1*) (NM_000033.4) that had been identified using whole-exome sequencing for the genome of the affected members (II-1, II-3, and II-6). The same variant was detected in a heterozygous state in the asymptomatic female carrier (II-8). **(B)** Wild-type and mutant residues (p.Tyr296Cys) in the ABCD1 protein are colored light-green and represented as sticks alongside the surrounding residues, which were involved in any interaction type. Red dots are hydrogen bonds, with orange representing weak ones. The crystal structure of the domain from wild-type ABCD1 isoform 1 was generated using SWISS-MODEL (https://swissmodel.expasy.org/) and is depicted as a cartoon representation. **(C)** Results obtained using other predictive tools (NMA based and other structure-based approaches) are also displayed, which predict the effect of the mutation using the DynaMut web-server with the normal mode analysis function (http://biosig.unimelb.edu.au/dynamut/). Visual representation of the Δ vibrational entropy energy in which the amino acids are colored according to the vibrational entropy change upon mutation. Blue regions indicate rigidification and red regions indicate an increase in flexibility.

The whole-exome sequencing results revealed that the affected family members carried a hemizygous missense variant (c.887A>G, p.Tyr296Cys) of *ABCD1*, which was confirmed by Sanger sequencing (Figure 3A). The same variant was detected in a heterozygous state in the asymptomatic female carriers (I-2 and II-8). This variant has been previously reported in patients with X-ALD, and classified as pathogenic according to the ACMG criteria, but no functional evidence for this variation was found in the ClinVar database (16, 17).

The p.Tyr296 residue is located on ABC transporter transmembrane region 2. Protein crystallization revealed that the p.Tyr296Cys variant might affect the formation of weak hydrogen bonds between the surrounding residues (Figure 3B). Moreover, the p.Tyr296Cys variant was found to be causing changes in the vibration-related entropy change upon mutation, increasing molecular flexibility (ΔΔS_vib_ ENCoM: 0.121 kcal.mol^−1^.K^−1^), which might affect switching mechanism for providing different kinetic control for different transporters (Figure 3C).

## Discussion

All of the affected members in this family presented with slurred speech, ataxia, and spasticity with or without paraplegia, which were similar to the clinical manifestations of spastic ataxia or spinocerebellar ataxia. Although there were different findings on brain MRI among the affected members, an ALD diagnosis was suspected based on high VLCFA levels in the plasma, and finally confirmed by the detection of the *ABCD1* mutation.

ALD has a wide range of phenotypes according to the involved lesions (1). The cerebello-brainstem dominant form mainly involves the cerebellum and brainstem, and has been referred to by various names such as spinocerebellar variant, olivopontocerebellar form, and ataxic variant (3–12). This phenotype has been rarely reported in the literature, and is estimated to account for 1–2% of ALD cases. However, it may be underdiagnosed because the clinical features can resemble those seen in patients with MSA, ILOCA, and MS. Indeed, some patients who received antemortem clinical diagnose of olivopontocerebellar atrophy, MSA, MS, or schizophrenia were diagnosed with ALD at autopsy (3, 9–11). According to a review of 34 cases with the adult-onset cerebello-brainstem dominant form of ALD, the age at onset was 33 ± 11 years (mean ± SD), which was younger than that of MSA or ILOCA (3). The common clinical manifestations were cerebellar ataxia, gait disturbance, slurred speech, and pyramidal signs. About half of the patients had a family history of ALD or Addison's disease. VLCFA levels were elevated in all of these patients, and more than two-thirds of them presented adrenal insufficiency. T2-weighted hyperintensities in the internal capsule, brainstem, and/or cerebellum might be helpful in diagnosing this rare phenotype, but some patients have been reported with brainstem or cerebellar atrophy but without typical T2-weighted hyperintensities on MRI.

Despite the presence of the same *ABCD1* mutation, there could be several different phenotypes within a family (13–15). A literature review found that about 38% of sibling pairs presented with different clinical types of ALD, and the degree of similarity between ages at onset was 55–60%, regardless of how closely related the family members were (13). In the present family, three affected members mainly showed the cerebello-brainstem dominant form of ALD, but had different clinical characteristics such as age at onset, disease severity, cognitive impairment, and motor weakness. The location and severity of T2-weighted hyperintensities also differed markedly among the family members. It was particularly interesting that the younger brothers (II-6 and II-8) displayed a far worse clinical phenotype than the proband (II-1) (the oldest brother) in terms of neurological symptoms and brain lesions, even though most of their VLCFA levels and ratios were lower than those of the proband. Therefore, it may be difficult to accurately reflect the severity of the disease based on only biochemical findings (1). The deceased member (II-5) was also suspected to have CCALD based on the early-onset and rapid neurological deterioration of their condition. Indeed, the same *ABCD1* mutation has been reported in other ALD phenotypes including CCALD, ACALD, and AMN (16, 17). The range of phenotypic expression, disease severity, and prognosis were unpredictably variable, and there were no obvious correlations between the phenotypes and genotypes of ALD. All of these findings suggest that modifier genes or epigenetic or environmental factors could underlie the high variability of clinical manifestations. A previous study found that decreased expression levels of another peroxisomal transporter gene (*ABCD4*) and VLCFA synthetase gene (*BG1*) tended to be correlated with disease severity in ALD (17). However, we could not detect any variant in modifier genes such as *ABCD2, ABCD3, ABCD4, BG1*, and *VLCS*. The molecular basis for the allelic heterogeneity of X-ALD is currently poorly understood. In order to explain the various clinical phenotypes in patients within the same household in detail, RNA sequencing-based transcriptome profiling analysis, bisulfite sequencing-based methylome profiling or ATAC-Seq-based open chromatin profiling analysis will be needed.

In conclusion, we have presented a cerebello-dominant form of X-ALD caused by a missense variant in *ABCD1*. This report highlights intrafamilial phenotypic variability in X-ALD, suggesting the existence of modifier genes or epigenetic or environmental factors. In addition, the cerebello-dominant form of X-ALD should be considered as a differential diagnosis of cerebellar ataxia, especially in cases with T2-weighted hyperintensities or atrophy in the brainstem or cerebellum on MRI.

## Data availability statement

The datasets presented in this study can be found in online repositories. The name of the repository and accession numbers can be found below: National Center for Biotechnology Information (NCBI) GenBank, https://www.ncbi.nlm.nih.gov/genbank/Banklt2611441, OP204635-OP204637.

## Ethics statement

All experiments followed the tenets of the Declaration of Helsinki, and written informed consent was obtained from the participants after the nature and possible consequences of this study had been explained. This study was approved by the institutional review boards of Pusan National University Yangsan Hospital (05-2022-157).

## Author contributions

J-HC contributed to the collection, interpretation of the data, and wrote the manuscript. HSK, JHL, and EHO contributed to the interpretation and analysis of data. CKC conducted the design and conceptualization of the study, interpretaion of the data, and revised the manuscript. All authors contributed to the article and approved the submitted version.

## Conflict of interest

The authors declare that the research was conducted in the absence of any commercial or financial relationships that could be construed as a potential conflict of interest.

## Publisher's note

All claims expressed in this article are solely those of the authors and do not necessarily represent those of their affiliated organizations, or those of the publisher, the editors and the reviewers. Any product that may be evaluated in this article, or claim that may be made by its manufacturer, is not guaranteed or endorsed by the publisher.
